# Performance of preoperative plasma tumor markers HE4 and CA125 in predicting ovarian cancer mortality in women with epithelial ovarian cancer

**DOI:** 10.1371/journal.pone.0218621

**Published:** 2019-06-20

**Authors:** Daniela Furrer, Jean Grégoire, Stéphane Turcotte, Marie Plante, Dimcho Bachvarov, Dominique Trudel, Bernard Têtu, Pierre Douville, Isabelle Bairati

**Affiliations:** 1 Université Laval Cancer Research Center 11, Côte du Palais, Quebec City, QC, Canada; 2 Research Center of the CHU de Québec-Université Laval (Oncology division), Côte du palais, Quebec City, QC, Canada; 3 Faculty of Medicine, Université Laval, Avenue de la Médecine, Quebec City, QC, Canada; 4 Gynecologic Oncology Division, Centre Hospitalier Universitaire (CHU) de Québec-Université Laval, L’Hôtel-Dieu-de-Québec, Québec, Québec, Canada; 5 Department of Pathology, Hôpital Saint-Luc, Centre Hospitalier Universitaire de Montréal, rue Saint-Denis, Montréal, Québec, Canada; The University of Texas MD Anderson Cancer Center, UNITED STATES

## Abstract

Clinical utility of new biomarkers often requires the identification of their optimal threshold. This external validation study was conducted to assess the performance of the preoperative plasma tumor markers HE4 and CA125 optimal cut-offs to predict cancer mortality in women with epithelial ovarian cancer (EOC). Participating women had upfront debulking surgery in the University Hospital of Quebec City (Canada) between 1998 and 2013. A total of 136 women participated in the training cohort (cohort 1) and 177 in the validation cohort (cohort 2). Preoperative plasma HE4 and CA125 levels were measured by Elecsys. Optimal thresholds were identified in the cohort 1 using time-dependent receiver operating characteristic (ROC) curves. Multivariate Cox models were used to validate the biomarkers using their optimal cut-offs in the cohort 2. The likelihood ratio (LR) test was done to test whether the biomarkers added prognostic information beyond that provided by standard prognostic factors. The Areas Under the Curves (AUC) for HE4 and CA125 were respectively 64.2 (95% CI: 54.7–73.6) and 63.1 (95%CI: 53.6–72.6). The optimal thresholds were 277 pmol/L for HE4 and 282 U/ml for CA125. Preoperative plasma HE4 (≥277 pmol/L) was significantly associated with EOC mortality (adjusted hazard ratio (aHR): 1.90; 95% CI:1.09–3.29). The prognostic effect of HE4 was strongest in the subgroup of women with serous ovarian cancer (aHR: 2.42; 95% CI: 1.25–4.68). Using a multivariate model including all standard prognostic factors, this association was maintained (aHR: 2.21; 95% CI: 1.15–4.23). In addition, preoperative plasma HE4 added prediction for death over the standard prognostic markers in women with serous tumors (p-value for LR-test: 0.01). Preoperative CA125 was not associated with cancer mortality, both in women with EOC and in those with serous tumors. Preoperative HE4 is a promising prognostic biomarker in EOC, especially in serous tumor.

## Introduction

In North America, ovarian cancer is the second most commonly diagnosed cancer of the female genital tract and the most lethal gynecologic cancer [1,2]. According to SEER data, most women (66%) with epithelial ovarian cancer (EOC) are diagnosed with advanced stages [3]. In Canada, statistics published in 2018 showed that 63% of women are diagnosed with advanced stages and that stage IV varied from 14.1% to 24.2% across the provinces [1]. The relative survival of women with EOC has improved since the 80s [4]. Yet, the ten-year relative survival rates for women with stages III and IV EOC are respectively 23% and 8% [3]. Several treatment strategies are presently emerging, in particular for patients with worse prognosis, using drugs targeting the vascular endothelial growth factor (VEGF) or poly (ADP-ribose) polymerase (PARP) inhibitors [5].

In this context, it is clinically relevant to identify additional biomarkers to improve outcome prediction in women with EOC. Human Epididymis Protein 4 (HE4), which is overexpressed in ovarian epithelial cancer cells, is presently one of these promising biomarkers. Studies reported good performances of circulating HE4 for ovarian cancer detection [6,7] and recent systematic reviews pointed out its promising role as a prognostic biomarker [8,9]. In addition, recent *in vitro* and *in vivo* studies are supporting the fact that HE4 plays a role in several molecular pathways associated with cell proliferation, tumor growth, and metastasis in ovarian cancer [10–12].

For decision-making, clinicians used to have valid biomarkers with optimal thresholds for predicting survival of women with EOC. In our previous work, we reported that a cut-off of 394 pmol/L based on the median value of the distribution of the preoperative plasma HE4 levels in our EOC population was able to predict cancer mortality [13]. In addition, our analyses did not support a linear functional relationship between HE4 and mortality, and suggested a plateau for highest HE4 values. The present study was conducted to establish and validate the optimal threshold of preoperative plasma HE4 levels for predicting EOC mortality. As Cancer Antigen 125 (CA125) is presently the most commonly used biomarker in EOC, we also investigated its performance for predicting EOC mortality using the same methodology.

## Materials and methods

### Study design

An external validation study [14] was conducted using two independent cohorts of women with EOC. The training cohort (cohort 1) was used to identify the optimal thresholds of HE4 and CA125, while the validation cohort (cohort 2) was used to validate the ability of the optimal cut-off for each biomarker to predict EOC-specific mortality.

### Patients

Eligible patients were all newly diagnosed women with EOC, treated surgically in the University Hospital of Quebec City, Canada. Women had to have an upfront debulking surgery, performed by trained gynecologic oncologists, complemented by adjuvant chemotherapy, when indicated. Women who received neoadjuvant chemotherapy, intraperitoneal chemotherapy, hyperthermic intra-peritoneal chemotherapy, or new therapeutic interventions such as anti-VEGF agents or PARP inhibitors were not eligible. Eligible women operated between January 1, 1998 and December 31, 2006 constituted the cohort 1 (n = 136) [13]. The cohort 2 (n = 177) was composed of eligible women who underwent surgery between January 3, 2007 and October 15, 2013. All women recruited in this study signed an informed consent form to participate in the “CHU-biobank”. The study protocol was approved by the Research Ethics Committee of the “CHU de Quebec-Université Laval”.

### Data collection

Age, clinical characteristics (FIGO stage, histology, grade, preoperative CA125) and treatment modalities (e.g. residual tumor, chemotherapy regimen) were collected from the patients’ medical records. Dates and causes of death were obtained from the Quebec mortality registry using the unique Quebec health insurance identifier (last record linkage July 30, 2015).

### Pre-operative HE4 and CA125 assessments

EDTA plasma samples were collected prospectively before surgery. Plasma aliquots were frozen and stored at -80°C. For both cohorts, plasma HE4 levels were quantified on the cobas e601 analyzer using an electro-chemoluminescence immunoassay (Elecsys HE4 kit, Roche Diagnostics, Laval, Canada), while preoperative plasma CA125 levels were measured using the Elecsys CA 125 II kit (Roche Diagnostics). Both assays were performed according to the manufacturer’s instructions. The laboratory technician who performed the HE4 and CA125 analyses was blinded to women’s outcomes and other clinical information.

### Statistical analysis

Standard statistic tests were done to compare the characteristics of the two cohorts. In both cohorts, medians and interquartile ranges (IQR) were generated to describe plasma HE4 and CA125 levels according to standard prognostic factors. Kruskal-Wallis tests were done to test whether plasma HE4 and CA125 levels differed according to levels of standard prognostic factors.

In the training cohort, we generated for each biomarker a time-dependent receiver operating characteristic (ROC) curve for right-censored data using the Inverse Probability of Censoring Weighted Estimator [15,16]. The outcome was the mortality by ovarian cancer at 5 years. The areas under the curves (AUC) and their 95% confidence interval (CI) were generated. The analyses were performed in R with the package “timeROC” developed by Blanche et al.[15]. For each biomarker, the optimal cut-off point was identified using the Youden index J [17].

In the validation cohort, we constructed multivariate Cox proportional hazard regression models to verify that each biomarker, defined in two categories according to their respective optimal threshold, was associated with EOC-specific mortality. We also tested whether each biomarker contributed additional information about death by ovarian cancer beyond the information provided by standard prognostic factors. When necessary, the Firth’s maximum likelihood estimation was done [18]. Specific survival was calculated from the date of surgery to the date of death by ovarian cancer or July 30, 2015 (last record linkage). According to the REMARK guidelines [19], two multivariate models were constructed: the standardized and the final models. The standardized model included all EOC standard prognostic factors: age (continuous), FIGO stage (III, IV vs II, I), histology (serous versus other types) and grade (1, 2, 3). Grade was assessed according to the Silverberg system [20]. In multivariate analyses conducted among the subgroup of serous tumors, grades 2 and 3 were grouped together since the Silverberg system correlates very well with the two-tier system [21]. The residual tumor was not considered in the standardized model, since this factor was highly associated with FIGO stage in our data (p≤0.0001 in both cohorts). All the standard prognostic factors (age, FIGO stage, histology and grade) were considered to construct the final model. A backward selection was done to include only standard prognostic factors significantly (p≤0.05) associated with death by EOC [22]. Only FIGO stage was retained in the final model. Of note, this model was identical to the final model already generated in the training cohort [13]. Finally, each biomarker (HE4, CA125) was added separately to the final and standardized models for testing whether the biomarker added prognostic information beyond that provided by standard prognostic factors. A Likelihood Ratio test was used to compare the standardized model including only standard prognostic factors to the standardized model which also included the biomarker of interest [22]. The same procedure was done with the final model. Finally, a subgroup analysis was done in women with serous tumors using the same thresholds for HE4 and CA125. Adjusted hazard ratios (HR), as well as their 95% confidence intervals (CI), were estimated. For all Cox models, the proportional hazards assumption was verified using the standardized score process and the overall adequacy of models was assessed by examining the deviance residuals. These statistical analyses were conducted using SAS 9.3 (SAS institute, Cary, NC). All statistical tests were two-sided.

## Results

At 5 years, there were 66 deaths from EOC in the cohort 1 (n = 136) and 77 in the cohort 2 (n = 177), respectively. EOC-specific mortality rates at 5-years were 53% (95% CI = 44–61%) in the cohort 1 and 54% (95% CI = 45–62%) in the cohort 2, respectively. Table 1 showed no statistical difference between the two cohorts for preoperative HE4 and CA125 levels, but there were differences for FIGO stage, grade, and residual tumor.

**Table 1:** Clinical characteristics of women with epithelial ovarian cancer.

Characteristics	Cohort 1	Cohort 2	P-value
	(N = 136)	(N = 177)	
Age (years)–mean (SD)	61.9 (11.1)	61.9 (10.1)	0.96
Age (years)–n (%)			
<55	40 (29)	46 (26)	0.23
55–74	76 (56)	114 (64)	
≥75	20 (15)	17 (10)	
FIGO Stage–n (%)			
I	14 (10)	37 (21)	0.02
II	12 (9)	22 (12)	
III	95 (70)	107 (61)	
IV	15 (11)	11 (6)	
Histology–n (%)			
Serous tumors	98 (72)	115 (65)	0.15
Clear cell tumors	13 (10)	21 (12)	
Endometrioid tumors	13 (10)	24 (13)	
Mucinous tumors	3 (2)	8 (5)	
Malignant Brenner tumor	1 (0)	0 (0)	
Undifferentiated carcinoma	0 (0)	3 (2)	
Transitional cell carcinoma	3 (2)	0 (0)	
Mixed Malignant Mullerian tumors	5 (4)	6 (3)	
Grade–n (%)			
1	17 (12)	17 (10)	<0.01
2	50 (37)	25 (14)	
3	69 (51)	135 (76)	
Preoperative plasma CA125 (U/ml)–median (IQR)	422 (175–874)	407 (92–1107)	0.27
Preoperative plasma HE4 (pmol/L)–median (IQR)	427 (185–1019)	304 (134–926)	0.11
Adjuvant carboplatin and paclitaxel–n (%)	100 (74)	143 (81)	0.13
Residual tumor- n (%)			
None	37 (27)	81 (46)	<0.01
< 2 cm	46 (34)	52 (29)	
≥ 2 cm	53 (39)	44 (25)	

IQR: Interquartile range (25^th^ and 75^th^ percentiles)

In both cohorts, preoperative plasma HE4 levels were significantly associated with standard prognostic factors (Table 2). In particular, HE4 levels increased significantly with FIGO stage, preoperative CA125, residual tumor, and in serous tumors.

**Table 2:** Preoperative plasma HE4 levels according to standard prognostic factors in women with epithelial ovarian cancer.

	Cohort 1 (N = 136)			Cohort 2 (N = 177)		
Factors	N	HE4 (pmol/L) Median (IQR)	P-value	N	HE4 (pmol/L) Median (IQR)	P-value
Age (years)						
<55	40	261 (113–481)	0.007	46	304 (94–546)	0.22
55–74	76	504 (234–1136)		114	292 (141–1083)	
≥75	20	611 (227–1604)		17	429 (155–820)	
FIGO stage						
I	14	153 (60–237)	0.0004	37	98 (78–240)	<0.0001
II	12	202 (106–439)		22	141 (76–298)	
III	95	495 (229–1163)		107	494 (212–1116)	
IV	15	501 (277–1820)		11	764 (303–2078)	
Histology						
Serous	98	552 (269–1221)	<0.0001	115	502 (180–1226)	<0.0001
Others	38	171 (81–398)		62	117 (78–318)	
Grade						
1	17	211 (132–269)	0.001	17	131 (94–203)	0.005
2	50	553 (315–1320)		25	400 (220–1116)	
3	69	387 (154–1068)		135	343 (139–971)	
Preoperative CA125						
1^st^ quartile	34	177 (81–301)	<0.0001	44	94 (76–145)	<0.0001
2^nd^ quartile	34	394 (163–714)		44	244 (141–435)	
3^rd^ quartile	34	531 (315–2316)		44	437 (265–731)	
4^th^ quartile	34	711 (434–2134)		45	1223 (672–2308)	
Residual tumor						
None	37	187 (68–363)	<0.0001	81	172 (87–318)	<0.0001
< 2 cm	46	364 (193–596)		52	468 (206–1161)	
≥ 2 cm	53	798 (417–2248)		44	688 (348–1301)	

IQR: interquartile range (25^th^ and 75^th^ percentiles)

Preoperative plasma CA125 levels also increased significantly with FIGO stage, residual tumor, and in serous tumors (Table 3).

**Table 3:** Preoperative plasma CA125 levels according to standard prognostic factors in women with epithelial ovarian cancer.

	Cohort 1 (N = 136)			Cohort 2 (N = 177)		
Factors	N	CA125 (U/ml) Median (IQR)	P-value	N	CA125 (U/ml) Median (IQR)	P-value
Age (years)						
<55	40	455 (138–832)	0.83	46	353 (75–1015)	0.78
55–74	76	433 (192–1105)		114	401 (108–1137)	
≥75	20	417 (142–1506)		17	474 (259–617)	
FIGO stage						
I	14	123 (70–202)	0.0003	37	90 (42–552)	<0.0001
II	12	377 (135–1040)		22	104 (42–350)	
III	95	515 (256–1113)		107	545 (216–1548)	
IV	15	457 (149–1442)		11	1112 (386–1762)	
Histology						
Serous	98	552 (256–1113)	0.0004	115	554 (200–1480)	<0.0001
Others	38	191 (115–610)		62	123 (42–436)	
Grade						
1	17	140 (126–330)	0.01	17	91 (52–431)	0.07
2	50	434 (256–762)		25	419 (182–1137)	
3	69	562 (168–2067)		135	431 (110–1107)	
Residual tumor						
None	37	147 (86–345)	<0.0001	81	144 (56–762)	<0.0001
< 2 cm	46	512 (235–1186)		52	550 (327–1500)	
≥ 2 cm	53	601 (291–1113)		44	582 (254–1687)	

IQR: Interquartile range (25^th^ and 75^th^ percentiles)

**Fig 1:**
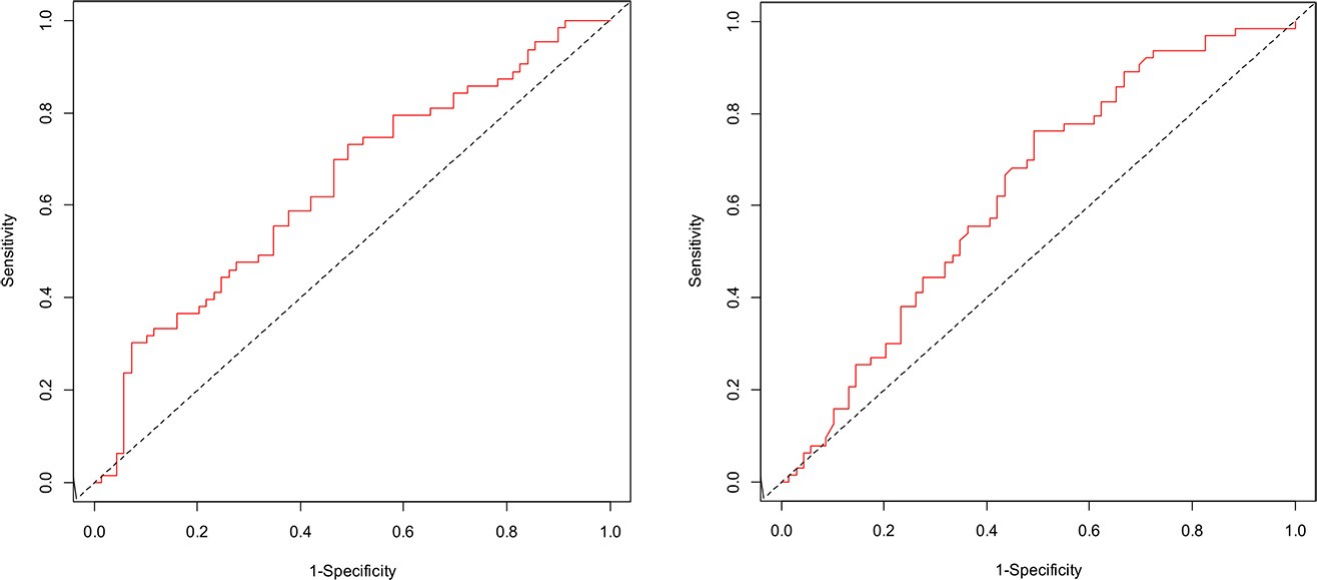
Receiver Operating Characteristic (ROC) curve for prediction of EOC death at 5-years in cohort 1. The left figure shows the ROC curve for prediction of EOC death by preoperative plasma HE4 levels (AUC = 64.2; 95% CI: 54.7–73.6). The right figure shows the ROC curve for prediction of EOC by preoperative plasma CA125 levels (AUC = 63.1; 95% CI: 53.6–72.6).

In the training cohort (cohort 1), the ROC curves showed a similar performance for HE4 and CA125 (Fig 1). All ROC curves differed significantly from 50%. The AUC for preoperative plasma HE4 was 64.2 (95% CI: 54.7–73.6) and the AUC for preoperative plasma CA125 was 63.1 (95% CI: 53.6–72.6). The optimal threshold was 277 pmol/L for HE4 (sensitivity, 0.75; specificity, 0.49) and 282 U/ml for CA125 (sensitivity, 0.76; specificity, 0.51), respectively.

In the validation cohort (cohort 2), we validated the use of the preoperative plasma HE4 and CA125, defined according to their optimal cut-offs, for predicting EOC-specific mortality. In the final model, the preoperative plasma HE4 defined according to its optimal cut-off (277 pmoL) was significantly associated with mortality by EOC (adjusted HR = 1.90; 95% CI: 1.09–3.29) (Table 4). In the standardized model, taking into account all the preoperative standard prognostic factors regardless of their statistical association with survival, the magnitude of the association between HE4 and EOC mortality decreased and was no longer statistically significant (adjusted HR = 1.61; 95% CI: 0.92–2.80). Using the preoperative CA125 with its optimal threshold of 282 U/ml in the standardized model, the association between CA125 and mortality by EOC was 1.36, but did not reach the statistical significance.

**Table 4:** Adjusted hazard ratios (HR) of death and their 95% confidence interval (CI) associated with preoperative plasma HE4 and CA125 in women with epithelial ovarian cancer (N = 177).

Factors	Final Models			Standardized Models		
	Without the Biomarker HR (95% CI)	With HE4 ≥ 277 pmol/L HR (95% CI)	With CA125 ≥ 282 U/ml HR (95% CI)	Without the Biomarker HR (95% CI)	With HE4 ≥ 277 pmol/L HR (95% CI)	With CA125 ≥ 282 U/ml HR (95% CI)
FIGO Stage						
(III-IV) vs (I-II)	8.08 (3.51–18.62)	6.11 (2.57–14.55)	7.04 (2.99–16.54)	6.93 (2.87–16.78)	5.32 (2.13–13.30)	5.72 (2.28–14.37)
Age						
(continuous)				1.01 (0.99–1.03)	1.01 (0.99–1.03)	1.01 (0.99–1.03)
Histology						
(Others vs serous)				1.39 (0.71–2.72)	1.30 (0.66–2.54)	1.33 (0.68–2.59)
Grade						
(2 vs 1)				8.59 (0.50–146.96)	6.60 (0.38–114.88)	7.15 (0.42–122.31)
(3 vs 1)				10.02 (0.62-162-48)	7.83 (0.48–129.00)	8.92 (0.56–143.05)
Biomarker		1.90 (1.09–3.29)	1.47 (0.87–2.48)		1.61 (0.92–2.80)	1.36 (0.79–2.34)
LR test statistic (P-value)		5.72^a^ (0.02)	2.21^a^ (0.14)		3.07^b^ (0.08)	1.22^b^ (0.27)

^a^ The Likelihood Ratio (LR) test comparing the model including the biomarker to the final model without the biomarker.

^b^ The Likelihood Ratio (LR) test comparing the model including the biomarker to the standardized model without the biomarker.

In women with serous ovarian cancer, the magnitude of the association between preoperative plasma HE4 and mortality by ovarian cancer was strongest (adjusted HR = 2.42; 95% CI: 1.25–4.68) than among all EOC (Table 5). Furthermore, this association remained statistically significant (adjusted HR = 2.21; 95% CI: 1.15–4.23) in the standardized model, which included all the standard prognostic variables. The comparison of the Cox model including all standard prognostic factors (the standardized model) with the standardized model that additionally included the HE4, showed a statistically significant additional effect of HE4 on the prediction of mortality by ovarian cancer (LR test = 6.69; p-value = 0.01). In this subgroup, preoperative CA125 remained not associated with death by ovarian cancer.

**Table 5:** Adjusted hazard ratios (HR) of death and their 95% confidence interval (CI) associated with preoperative plasma HE4 and CA125 in women with serous ovarian cancer (N = 115).

Factors	Final Models			Standardized Models		
	Without the biomarker HR (95% CI)	With HE4 ≥ 277 pmol/L HR (95% CI)	With CA125 ≥ 282U/ml HR (95% CI)	Without the biomarker HR (95% CI)	With HE4 ≥ 277 pmol/L HR (95% CI)	With CA125 ≥ 282 U/ml HR (95% CI)
FIGO Stage						
(III-IV) vs (I-II)	17.09 (2.36–123.58)	12.58 (1.72–92.08)	16.85 (2.33–121.93)	7.60 (1.54–37.48)	6.21 (1.25–30.88)	7.14 (1.44–35.48)
Age						
(continuous)				1.01 (0.98–1.03)	1.01 (0.98–1.03)	1.01 (0.99–1.03)
Grade						
(2–3 vs 1)				3.12 (0.19–51.55)	2.08 (0.12–36.75)	2.90 (0.17–50.39)
Biomarker		2.42 (1.25–4.68)	1.56 (0.87–2.79)		2.21 (1.15–4.23)	1.48 (0.83–2.65)
LR test statistic (P-value)		8.21^a^ (0.004)	2.39^a^ (0.12)		6.69^b^ 0.01	1.89^b^ 0.17

^a^ The Likelihood Ratio (LR) test comparing the model including the biomarker to the final model without the biomarker.

^b^ The Likelihood Ratio (LR) test comparing the model including the biomarker to the standardized model without the biomarker.

## Discussion

In the training cohort of 136 women with EOC, both preoperative plasma HE4 and CA125 showed a good performance for identifying women at high risk of death by EOC. Optimal cut-offs were identified for the two markers measured before treatments: 277 pmol/L for HE4 and 282 U/ml for CA125. The external validation conducted in an independent cohort of 177 EOC women confirmed that women with preoperative plasma HE4 levels above the optimal cut-off were significantly at higher risk of death by EOC. This association was strongest in the subgroup of women with serous tumors. In addition, plasma HE4 levels defined according to the threshold of 277 pmol/L added prediction over the standard prognostic markers of death by ovarian cancer in the subgroup of serous carcinoma. Using the same methodology, we showed that preoperative plasma CA125 levels of at least 282 U/ml was not significantly associated with death by ovarian cancer both in EOC women and in those with serous tumors.

To our knowledge, this is the first study that identified and validated an optimal cut-off of preoperative plasma HE4 levels for predicting EOC mortality. Most studies reporting significant associations between higher preoperative HE4 levels and mortality have defined high and low levels of HE4 according to the median of the HE4 distribution in their EOC populations [13,23,24]. In these studies, the preoperative HE4 median values were about 400 pmol/L. Most of the studies that have used a standard HE4 cut-off of 70 pmol/L, as usually proposed for cancer detection, or a continuous log-transformed HE4 variable failed to detect an association between presurgical HE4 levels and survival [25–28]. In the first case, we could easily anticipate that the optimal HE4 threshold for EOC prediction would be higher than the threshold for EOC detection. In the second case, the absence of association between HE4, when defined as a continuous variable, and mortality might be partly explained by a non-linear functional relationship between HE4 and mortality, as observed in our training cohort.

Most of the studies that have identified optimal cut-offs of preoperative HE4 for cancer prediction have only considered short-term outcomes. Studies showed that HE4 has a good performance for predicting women with advanced EOC who could benefit from upfront optimal debulking surgery [29–31]. In these studies, the performance of the preoperative biomarkers, tested with the AUC, was consistently higher for HE4 than for CA125, but the difference between the two biomarkers did not reach the statistical significance when tested. The best cut-off levels of preoperative HE4 for predicting optimal cytoreductive surgery varied from 262 pmol/L to 473 pmol/L. In studies including all FIGO stages, the optimal HE4 cut-off levels were lower, around 220–235 pmol/L [32,33]. Noteworthy, Nassir et al. [34] evaluated the performance of preoperative HE4 and CA125 for predicting recurrence or death during the year following the end of the first-line chemotherapy in 79 EOC women with FIGO stages II-IV. The AUCs were 0.658 (95% CI: 0.535–0.781, p = 0.016) for HE4 and 0.62 (95%CI: 0.506–0.74, p = 0.046) for CA125, which is consistent with our study results. Nassir et al. [34] also identified an optimal cut-off of 165 pmol/L (sensitivity: 86.1, specificity: 34.9) for HE4, a value slightly lower to that detected in our study (277 pmol/L), while the optimal cut-off value for CA125 was 400 U/ml (sensitivity: 80.7; specificity: 0.50). However, none of the optimal HE4 cut-offs proposed in these studies was validated in external cohorts.

In this study, preoperative plasma HE4 levels were positively associated with all EOC standard prognostic factors in both cohorts, a finding which is consistently reported in others studies [23,24,27,28]. This study showed that preoperative plasma HE4, defined according to the optimal cut-off of 277 pmol/L was associated with EOC mortality in the model adjusting only for prognostic factors significantly associated with death. However, in the standardized model which included all standard prognostic factors regardless of their statistical significance, the magnitude of this association decreased and was of the borderline significance (p = 0.09). This latter result could be partly explained by the use of a more stringent model, which ensured a better control of potential confounders than in the final model, but with the inconvenience of decreasing the statistical power. In women with serous ovarian cancer, the associations remained statistically associated with mortality by ovarian cancer in both models. In addition, preoperative HE4 added prognostic information beyond that provided by standard prognostic factors. This means that this optimal HE4 threshold provides clinicians with prognostic information already before surgery in the subgroup of serous tumors.

Using the same methodology, we were not able to validate the preoperative CA125 cut-off of 282 U/ml for predicting mortality. In other studies, preoperative CA125 levels were consistently found associated with standard prognostic factors, such as observed in our study [23,35,36]. Few studies have assessed the prognostic significance of preoperative CA125. In a cohort study, including 940 EOC women mainly with FIGO stage I (80%), the AUC of preoperative CA125 for predicting overall mortality at 5 years was 0.63 (95% CI: 0.58–0.73), which is close to that observed in our study. The optimal CA125 cut-off for predicting mortality was 50 U/mL, but the validation of this cut-off was done on the same cohort [35]. Based on pretreatment CA125 values of 1,132 patients with advanced EOC treated by cytoreduction followed by six cycles of paclitaxel and cisplatin, Zorn et al. [36] showed that CA125 can be used to predict disease progression (recurrence or death). Using six predefined categories of CA125 (with unequal distance), the survival analyses indicated that the risk of disease progression increased with increased CA125 levels. However, Cox multivariate analyses showed that the risk of disease progression was statistically significant for the CA125 categories above 100 U/mL.

This study presents several strength and limitations. The methodology used in this study supports the validity of these results. First, the performance of the biomarkers was determined using ROC curves developed for longitudinal studies with censored data. This allows identifying the performance of preoperative HE4 and CA125 on long-term outcomes, such as death, a robust outcome in oncology. Second, our results were validated using an external cohort of women with EOC, which represents the gold standard. This approach is recommended for avoiding the overestimation of the biomarker performance usually observed in studies using internal validation [19]. One possible limitation in our study design is that the two cohorts were recruited from the same institution during different time periods. Several prognostic factors, such as FIGO stage, were not similarly distributed between the two cohorts. Cohort 2 presented a more favorable prognostic profile, with in particular less advanced cancer than in cohort 1. This suggests a better detection of early stage cancer in the most recent cohort since no neoadjuvant therapies were given in both cohorts. Strict eligibility criteria concerning treatment modalities in this study ensured that women received comparable management in the two cohorts. Upfront cytoreductive surgery was done according to the guidelines by trained gynecologic oncologists [37,38] and most of the women received the standard first-line chemotherapy consisting of carboplatin and paclitaxel in the two cohorts. However, women in the most recent cohort tended to receive more frequently the carboplatin-paclitaxel regimen and had less residual tumors. Despite these differences, the optimal threshold of preoperative plasma HE4 levels identified in the training cohort was able to differentiate long-term prognosis of women with EOC, as well in the subgroup of women with serous tumors, in the validation cohort. This suggests that this optimal HE4 cut-off is robust and could be applied in other populations, particularly in women with serous carcinoma.

Preoperative plasma HE4 levels might be useful for the clinical management of women with EOC, as it might identify women with worse prognosis. A better knowledge of women’s risk profile could contribute to the improvement of their follow-up. Additional prognostic information might help clinicians to better tailor treatment strategies, such as the use of neoadjuvant chemotherapy and of emerging therapies. However, additional studies are needed to evaluate the clinical utility of HE4 with the new therapies.

## Conclusions

Preoperative plasma HE4 is a promising prognostic biomarker in women with EOC, particularly in women with serous tumor. Using an external validation study, an optimal HE4 cut-off of 277 pmol/L before surgery was associated with mortality by ovarian cancer in both women with EOC and in those with serous tumor. In addition, in women with serous tumor, preoperative plasma HE4 adds prognostic information beyond that provided by standard prognostic factors. However, further studies are needed to re-validate this preoperative optimal HE4 cut-off in large cohorts and to test the validity of this cut-off in subgroups of women with EOC.
